# Additional Observations on the Invariable Existence of a Premonitory Diarrhœa in Cholera

**Published:** 1856-03

**Authors:** David Macloughlin

**Affiliations:** London, England. 34 Bruton Steet, Berkeley Square


### SELECTIONS.

*Additional Observations on the Invariable Existence of a Premonitory Diarrhœa in Cholera; being a reply to Dr. Hutchison's Article of September*, 1855. By DAVID MACLOUGHLIN, M. D., of London, England.

In the *New York Journal of Medicine*, for September last, there is an article by Dr. Hutchinson, of Brooklyn, to which, for the interest of Medical science, I wish to reply, and I trust that my reply will find a place in that valuable periodical.

Before going further, permit me to assure Dr. Hutchinson that I have had, and that I can have, no intention "of omitting" anything he has said on the question before us. My object is the advancement of medical science, and not "to gratify" myself, as well as others who rely on my statements more than on their own experience, by laying down rules which are not based on accurate observations and on accurate researches, at the bedside.

What are the points at issue between Dr. Hutchison and myself?

I have laid it down as an invariable rule, that cholera, that is, vomiting, spasms, etc., is always preceded by a diarrhœa, for a few hours, or for a few days, or for a few weeks.

In opposition to this, Dr. Hutchinson says:

1. That a person in good health, and consequently free from diarrhœa, may be seized simultaneously with vomiting, spasms, purging, etc.

2. That a person in good health, and consequently free from diarrhœa, may be seized simultaneously with vomiting, purging, collapse, etc., without having had any spasms.

I will not appeal, for reasons which will be at once understood, to my own private and individual observations and researches at the bed-side, to prove that every case of cholera is preceded by a diarrhœa, and that the rule I have laid down is correct; but I will appeal to the observations, and to the researches at the bed-side, of gentlemen holding public appointments, and who, consequently, have carried on their observations and their researches publicly, and who have taken the trouble to certify to the General Board of Health here, that every case of cholera admitted into their establishments had had a premonitory diarrhœa, for a longer or shorter period, previous to the attack of vomiting, spasms, etc., and I beg to name the Medical Staff of the Middlesex, the St. Thomas, the Westminster, the Homœopathic, the St. Mary's, the University College Hospital, and the St. Bartholomew Hospital, as the gentlemen who have given the above certificates to the General Board of Health.

I must add, also, that six and thirty medical gentlemen employed at the Popular Union, and who have kept a valuable record of all of the cholera cases which have occurred for the last five years, in that Union, have certified that every case had had a diarrhœa, for a shorter or longer period, previous to the attack of vomiting, spasms, etc. And further, by referring to the Registrar General's Return of Births and Deaths, for 1853, and by referring to the *Medical Times* for the year 1853, it will be found that every case of death from cholera in London, during 1853, had had a diarrhœa before being attacked with vomiting, spasms, etc. Consequently, with this mass of evidence before me, I must conclude that I am right in having laid it down as a rule, that every case of cholera is preceded by a diarrhœa for a few hours, or for a few days, or for a few weeks, and, consequently, I submit, this is an answer to Dr. Hutchinson's first objection, viz: that a person in perfect health, and consequently free from diarrhœa, may be seized simultaneously with vomiting, spasms, purging, etc.

It seems that Dr. Hutchinson places implicit confidence in the statement of his patients, that they were seized simultaneously with vomiting, spasms, purging, etc.

If the Doctor will take the trouble to refer to page 207 of this Journal, for September, he will find it reported by Dr. Vanderveer, in his excellent article on cholera, that cases were admitted into his wards in a dying condition, who neither vomited nor purged after admission, and their relations who accompanied them, stated positively, that they had not previously; but what did the autopsy reveal? that not a particle of food could be discovered in the stomach, or a particle of fæcal matter could be discovered in the intestines, showing that these patients had both vomited and purged severely before being admitted into the Hospital.

Therefore, the statements of patients, and the statements of their friends, are not to be implicitly depended on—and we must remember that, at the bed-side, we must be guided in forming our opinion on the case by our knowledge of anatomy, physiology, and pathology, and that we must not blindly trust to the word of the inhabitant of a palace or to the word of the occupier of a hovel.

As to Dr. Hutchinson's second objection:—that a person in perfect health, consequently free from diarrhœa, may be seized simultaneously with vomiting, purging, collapse, etc., without having had any spasms. Again, I will not appeal to my private and individual observations and researches, in opposition to Dr. Hutchinson's statement on this point, but I will refer to the observations and to the researches of the medical staff of the above seven Hospitals, and to the observations and researches of the six and thirty medical gentlemen employed at the Popular Union, and further, to the Registrar General's weekly Return of Births and Deaths for 1853, and to the *Medical Times* for 1853, who all admit that spasms are one of the invariable symptoms of cholera, and mark the ending of the premonitory stage and the beginning of the second stage of cholera.

But is it not well known to the profession that spasms are so invariable a symptom of cholera—that, even for hours after death, we often see the extremities suddenly jerked up by spasms, to the great alarm of all who are not aware of this pathological fact? Consequently, we have, as yet, no authenticated case of cholera without spasms, and it must remain as an admitted fact, that spasm is an invariable symptom of cholera.

At page 225 of this Journal for September, Dr. Hutchinson says: "That, according to Dr. Macloughlin, diarrhœa becomes cholera as soon as cramps are developed, and not till then."

If Dr. Hutchinson will take the trouble to refer to the preface of the "*Result of an Inquiry into the Invariable Existence of a Premonitory Diarrhœa in Cholera*," which is in his hands, he will there see that it is said, that cholera has four stages—that of diarrhœa—that of vomiting, spasms, etc.—that of collapse—and that of reaction; and, therefore, Dr. Hutchinson is in error when he says, "That, according to Dr. Macloughlin's own showing, diarrhœa becomes cholera only as soon as cramps are developed, and not till then."

As to the fact that a person, laboring under painless diarrhœa, may be walking about for amusement, or for business, or engaged at his usual occupations at home—with the cold cyanic state of the skin, and with the cold clammy dew of death all over his body—the heart having ceased to beat, and the blood having ceased to circulate—unconscious that he has anything serious the matter with him, which is doubted by Dr. Hutchinson, I must again refer to the observations of all those gentlemen who have seen, and who have minutely studied, the rise and progress of cholera, at the bed-side, in all parts of the world; and it will be found that they have met with cases where the patient was laboring under painless diarrhœa, who was pulseless, and where the beating of the heart could not be heard, and who had the cold cyanic state of the skin, with the cold clammy dew of death all over their body, and who were still walking about, and complaining of no pain or uneasiness, and the next moment, possibly, were prostrated by vomiting, spasms, etc.

In two such cases, in 1832, when bleeding was supposed to be a cure for cholera, according to Broussais' views, I opened the temporal and the brachial artery, in the same individual; and, although both patients lived for several hours after, without having the vessels secured, no blood flowed.—(See my letter to Dr. Cormack, Editor of the *Association Medical Journal*.)

Consequently, I submit that I am justified in stating, that a person having a painless diarrhœa, may have almost the whole serum of his blood drained away, his blood may have ceased to circulate, and his heart may have ceased to beat, although he may be walking about, unconscious that he has anything serious the matter with him, with his skin cyanic, and the cold dew of death upon him.*

In conclusion, I beg leave to say:

1. That cholera—that is, vomiting, spasms, etc.—is always preceded by a diarrhœa, for a few hours, or for a few days, or for a few weeks.
2. That there is, as yet, no well-authenticated case of cholera without an attack of spasms.

---

*In a pamphlet on the *Premonitory Symptoms of Cholera*, by Dr. Macloughlin, we find a similar statement: "We must not lose sight of this most important pathological fact, that it has been reserved to Cholera to show us that we can walk about for pleasure, or business, or attend to any laborious occupation we may have at home, after our blood has been drained by a painless diarrhœa of almost the whole of its serum, and after our blood has ceased to circulate, and after our heart has ceased to beat." No fact, case, or proof whatever, is given to substantiate this extraordinary assertion, which we need not say, is in violation of reason not less than every known physiological law. Whatever Cholera may have shown to London practitioners, it certainly has never exhibited on this side of the Atlantic such an anomaly as a person "walking about for pleasure, or business" with a lifeless, pulseless heart in his breast. We trust the author does not think us so credulous as to sanction such fabulous statements.—(*Eds. N. Y. Jour. of Med.*

3. That a painless diarrhœa may drain away almost the whole serum from the blood, until the blood has ceased to circulate, and until the heart has ceased to beat, before the patient is attacked with vomiting, spasms, etc., that is with the second stage of cholera.

4. That if the disease is scientifically attended to in its diarrhœal stage, it can be cured, and, consequently, the developed stage prevented, and life saved.

5. That these two pathological facts are now acquired to medical science, viz. : 1. That every case of cholera is preceded by a diarrhœa, for a few hours, a few days, or a few weeks. 2. That the disease, scientifically treated in the diarrhœal stage, is easily cured.

6. That it is in the power of human foresight to prevent an attack of developed cholera.

In a letter dated 2nd July, 1855, inserted in the *Association Medical Journal*, it was attempted to point out what diarrhœa, if left to itself, will run into cholera in a few hours; and what diarrhœa, if left to itself, will not run into cholera for a few days, or for a few weeks.

I am fully aware that this pathological point requires to be further studied. I, therefore, submit it to the profession, in the hope that more attentive observers will be enabled to give us valuable information on this important subject, so that the medical attendant, on arriving at the bedside, may be enabled to pronounce whether his patient will be attacked in a few hours, or in a few days, or in a few weeks, with cholera, if the diarrhœa is not scientifically attended to, or whether the diarrhœa will be cured by the efforts of nature; and so that the medical attendant may not be left, as he now is left, to establish his prognosis on conjectures.

There is, also, another point to which I beg leave to call the attention of the profession.

I have now seen five severe outbreaks of epidemic cholera, and I have reason to believe that a great and an important change takes place in the constitution of every individual, where epidemic cholera is about to break out, which change in the constitution of every individual, persists while the disease rages, and after the disease has passed away for some time.

That this change in the constitution of individuals is manifest by the facts, that those of a costive habit, who have a passage from their bowels only every two, three, four, or five days, of hard fæcal matter, have now a passage from their bowels daily, of soft fæcal matter.

That those who are in the habit of having a passage daily, of solid fæcal matter, have now two or three passages daily, from their bowels, of soft fæcal matter.

That those who usually require laxatives to keep their bowels free, now do not require laxatives; or, if they take any, they find that one-half, or one-quarter, the usual dose has the same effect as a full dose had formerly.

And, further, that it is now dangerous to give a full dose of purgative medicine, lest this dose should induce diarrhœa, followed, too often, by fatal cholera. The *Medical Times*, of September, 1854, page 272, contains the report of four cases of cholera, which were induced in St. George's Hospital here, by the administration of the full dose of purgative medicine, and of which, one died.

That, further, every individual in the locality is troubled with more flatus than usual, especially between one and five in the morning, and that every one about to be attacked with diarrhœa has a pressure, and a weight on the sphincter of the anus, and a feeling of insecurity, as if, at any moment, he would lose command over it.

If, in consequence of the observations and of the researches of the profession in every country, it is ascertained that this change in the constitution of all the individuals in a locality where cholera is about to break out, does take place, and that this change persists while cholera rages, and after it has passed away some time, it may throw some light on the etiology of the disease, and prove that cholera is not a contagious diease.—*N. Y. Jour. of Medicine.*

34 Bruton Steet, Berkeley Square, Oct. 28th, 1855.

